# Fibromyalgia pathogenesis explained by a neuroendocrine multistable model

**DOI:** 10.1371/journal.pone.0303573

**Published:** 2024-07-11

**Authors:** Ilaria Demori, Serena Losacco, Giulia Giordano, Viviana Mucci, Franco Blanchini, Bruno Burlando

**Affiliations:** 1 Department of Pharmacy, DIFAR, University of Genova, Genova, Italy; 2 Department of Industrial Engineering, University of Trento, Trento, (TN), Italy; 3 Delft Center for Systems and Control, Delft University of Technology, Delft, The Netherlands; 4 School of Science, Western Sydney University, Penrith, Australia; 5 Department of Mathematics, Computer Science and Physics, University of Udine, Udine, Italy; Georgia State University, UNITED STATES

## Abstract

Fibromyalgia (FM) is a central disorder characterized by chronic pain, fatigue, insomnia, depression, and other minor symptoms. Knowledge about pathogenesis is lacking, diagnosis difficult, clinical approach puzzling, and patient management disappointing. We conducted a theoretical study based on literature data and computational analysis, aimed at developing a comprehensive model of FM pathogenesis and addressing suitable therapeutic targets. We started from the evidence that FM must involve a dysregulation of central pain processing, is female prevalent, suggesting a role for the hypothalamus-pituitary-gonadal (HPG) axis, and is stress-related, suggesting a role for the HP-adrenocortical (HPA) axis. Central pathogenesis was supposed to involve a pain processing loop system including the thalamic ventroposterolateral nucleus (VPL), the primary somatosensory cortex (SSC), and the thalamic reticular nucleus (TRN). For decreasing GABAergic and/or increasing glutamatergic transmission, the loop system crosses a bifurcation point, switching from monostable to bistable, and converging on a high-firing-rate steady state supposed to be the pathogenic condition. Thereafter, we showed that GABAergic transmission is positively correlated with gonadal-hormone-derived neurosteroids, notably allopregnanolone, whereas glutamatergic transmission is positively correlated with stress-induced glucocorticoids, notably cortisol. Finally, we built a dynamic model describing a multistable, double-inhibitory loop between HPG and HPA axes. This system has a high-HPA/low-HPG steady state, allegedly reached in females under combined premenstrual/postpartum brain allopregnanolone withdrawal and stress condition, driving the thalamocortical loop to the high-firing-rate steady state, and explaining the connection between endocrine and neural mechanisms in FM pathogenesis. Our model accounts for FM female prevalence and stress correlation, suggesting the use of neurosteroid drugs as a possible solution to currently unsolved problems in the clinical treatment of the disease.

## Introduction

Fibromyalgia (FM) is a syndrome characterized by a wide set of apparently uncorrelated symptoms, the most annoying of which is chronic, multi-focal pain. Other major symptoms include fatigue, sleep disorder, cognitive impairment, and depression [1]. It is a disabling condition with a worldwide prevalence of 1–5%, which makes it a significant health, social, and economic burden [2]. The clinical approach to FM raises a series of problems that originate from the absence of reliable knowledge about the pathogenesis mechanisms [3]. In addition, diagnosis is difficult and frequently delayed by years [4]. Despite repeated efforts to define a set of standard diagnostic criteria [3,5], FM diagnosis is made particularly hard by the lack of biomarkers and symptom overlapping with akin disorders, collectively called central sensitivity syndromes [6]. Because of these drawbacks patient management is disappointedly challenging. Limited rates of remission or improvement are achieved with pharmacological treatments, notably anticonvulsants and antidepressants [7]. Slightly better results are frequently obtained with physical treatments, exercise, or mind-body techniques [4].

The difficulties met with FM are linked to its obscure etiology, despite a wide set of hypothesized mechanisms of pathogenesis [8]. Hence, there is a strong urgency of disclosing the causes of the disorder. To this aim, we started from a few fundamental FM aspects, summarized as follows. First, there is a wide consensus on the idea that FM is a central disorder [9], and therefore, it must involve a dysregulation of some spinal and/or supraspinal network, including pain processing. Second, it is characterized by a significant female prevalence, with a worldwide average female:male ratio of 3:1, arriving locally to 10:1 [10,11]. This suggests that the hypothalamus-pituitary-gonadal endocrine (HPG) axis must have a role. Third, there is evidence that FM is a stress-related disorder [12]. Alteration of endocrine stress biomarkers in consolidated FM conditions has been questioned [13], but on the other hand, FM patients show hypothalamus–pituitary–adrenocortical axis (HPA) dysfunction [14,15]. Hence, it is conceivable that the HPA axis could be involved in the mechanism of pathogenesis. Consistently, there is strong evidence that physical and psychological stressful events, including early life traumatic experience, are FM predisposing factors [16,17].

We have previously proposed a mechanism leading to FM chronic pain based on a thalamocortical loop system [18]. This hypothesis was based on the following assumptions: (i) spinal pain processing can be excluded as a generator of the disorder because of the negligible efficacy of opioids in FM pain treatment [19,20]; (ii) the thalamus is the major supraspinal relay center in brain pain processing. In this study, we propose an extension of this model, by implementing together the above triad of FM basic elements, namely central network disorder, HPG axis, and HPA axis. We then show how the interplay between HPG and HPA axes, under peculiar combination of stress and gonadal conditions, can eventually produce weakening of GABAergic and strengthening of glutamatergic transmissions, thus switching the thalamocortical network to the pathological status.

## Methods

This is a theoretical study that presents a hypothesis about the biological mechanisms giving rise to a transition leading to the development of the FM syndrome. The hypothesis was based on a literature search on the PubMed database (National Institutes of Health, Bethesda, MD, USA), from inception through September 2023, combined with our previous thalamocortical loop model of FM chronic pain [18], and our review concerning FM in pregnancy [21]. The PubMed search was conducted by using as main keywords the names of different gonadal hormones, glucocorticoids, neurosteroids, and endocrine axes, variously combined with each other or with any of the following: “fibromyalgia”, “pain”, “glutamate”, “GABA”, and the names of various glutamate and GABA receptors.

The above literature data were used to build a graphical and mathematical description of an endocrine loop system representing the interaction between the HPG and HPA axes. Literature data were also used to infer possible repercussions of the endocrine system on the thalamocortical system, while the dynamics of the two systems were analyzed using MATLAB (version R2022a, MathWorks, Natick, MS, USA).

## Results

### Previous model: Thalamocortical loop system

#### Analysis of the system dynamics

**Fig 1:**
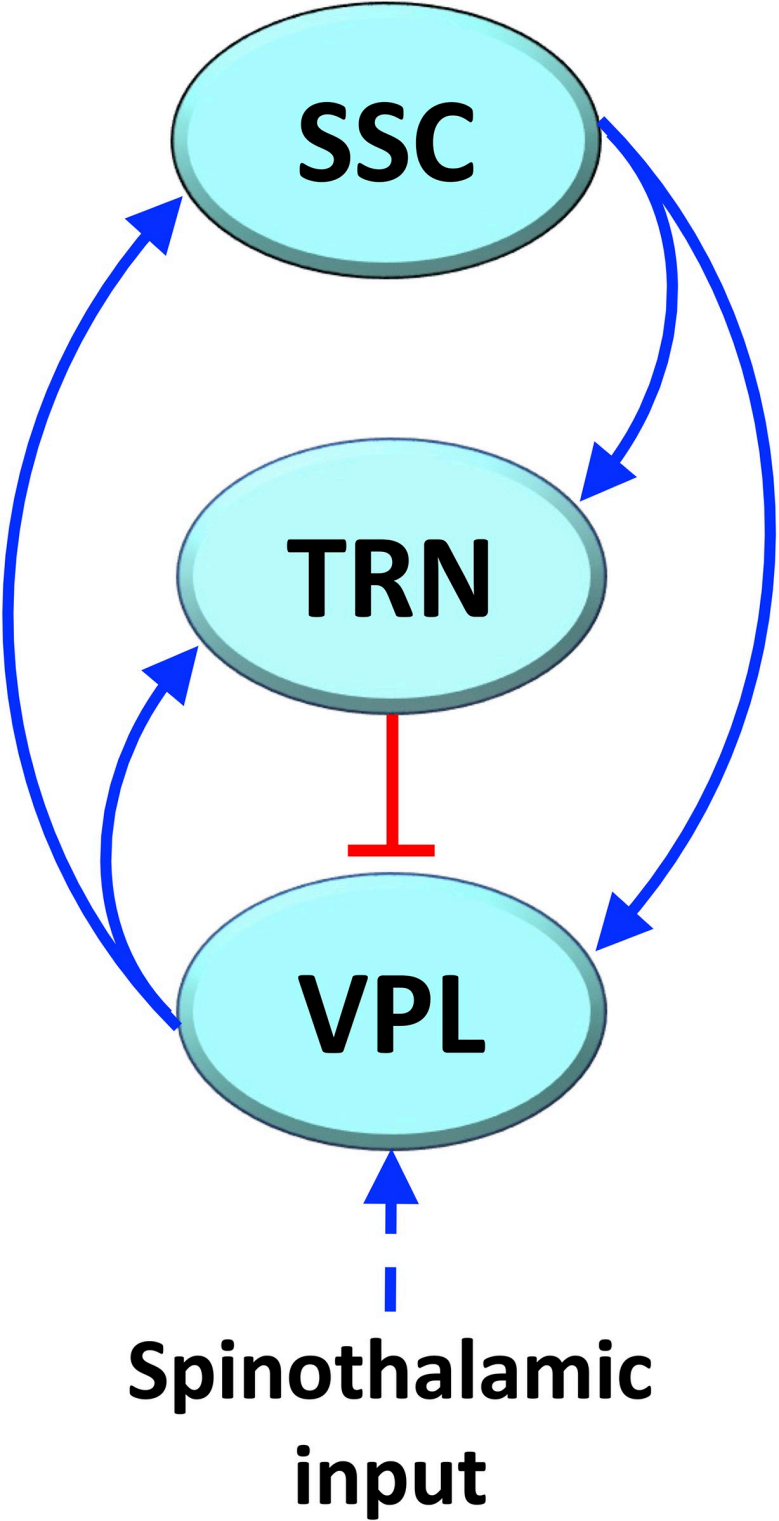
Simplified diagram of the thalamocortical loop considered in our previous FM model. Blue lines with arrowhead: excitatory glutamatergic connections; red lines with hammer endings: inhibitory GABAergic connections.

We have previously proposed that FM chronic pain depends on the malfunctioning of a thalamocortical loop network (Fig 1) [18]. The network is part of brain pain processing and is composed of the thalamic ventroposterolateral nucleus (VPL), the primary somatosensory cortex (SSC), and the thalamic reticular nucleus (TRN) [22]. Excitatory, first order glutamatergic fibers of the VPL projects to SSC and TRN; glutamatergic fibers of the SSC project to first order neurons of VPL and to TRN, and inhibitory GABAergic fibers of the TRN project to VPL (Fig 1) [23]. The SSC, despite its arrangement into distinct layers, is considered a single element in the loop given the excitatory coupling among layers [24,25].

The system consists of positive and negative loops, and we have demonstrated that it can globally behave as a monostable system (see Box 1) (when the negative loop is dominant) for stronger GABAergic transmission, or as a bistable system (when the positive loop is dominant) for a weakening of GABAergic transmission [18]. Bistable switches are known to promote predictable functional changes in living systems [26], also including pathogenesis processes [27].

**Box 1. Terms used in the analysis of dynamic systems**

**Table:** 

Dynamic system	A system that evolves over time, described in mathematical terms by a system of differential equations that represent the time evolution of the state variables in the *phase space*, where each axis is associated with a state variable and each point in the space represents a possible state configuration.
Steady states (equilibria)	Values of the state variables obtained by setting the time derivatives to zero in the system of differential equations and solving the corresponding algebraic system. If the system is at an equilibrium at a certain time, it will remain at the same equilibrium unless an external perturbation occurs.
Monostable, bistable, multistable systems	An equilibrium is *stable* if the system, after having moved away from this equilibrium due to a (sufficiently small) external perturbation, goes back to the equilibrium. Monostable systems admit only one stable equilibrium, bistable systems exactly two, and multistable systems more than two.
Bifurcation parameter	A parameter present in the differential equations that is varied when computing a bifurcation diagram.
Bifurcation diagram	It is the diagram that shows the values and the stability properties of the system equilibria for different values of a bifurcation parameter, and thus reveals whether a bifurcation (i.e., a change in the number of equilibria and/or in their stability properties) occurs. We call *bifurcation value* the value of the bifurcation parameter at which the system undergoes a bifurcation (for instance, transitioning from monostability to multistability).
Phase portrait	Graphical representation of the trajectories of a dynamic system during its time evolution starting from given initial conditions. Each point of a trajectory represents the values of the state variables (i.e., their Cartesian coordinates) at a different time instant.
Initial condition	It is the starting point of a trajectory, namely, the configuration in which the system happens to find itself at the beginning of its time evolution. In the analysis of dynamic systems, the initial conditions are typically assumed to be determined by factors that are external to the model (perturbing events).
Basin of attraction of a given steady state	Set of initial conditions starting from which the system trajectories reach the given steady state. Depending on the starting point, the system will converge to one of the stable steady states. Each steady state has its own basin of attraction and there is no overlapping among different basins of attraction. A system trajectory is attracted by a certain stable steady state when it starts sufficiently close to it (namely, within its basin of attraction).

We developed here a more complete analysis of the thalamocortical loop system by considering variations in strength of both GABAergic and glutamatergic transmission. In addition, the strength of the self-limiting term in the differential equations and its effect on the system dynamics were also considered. The dynamics of the thalamocortical loop system were mathematically described by the following system of differential equations [18]:

$$\tau \dot{S}+\theta S=f(bV)$$ (1)

$$\tau \dot{T}+\theta T=f(bV+bS)$$ (2)

$$\tau \dot{V}+\theta V=\frac{g(aT)∙{\left(bS\right)}^{h}}{{{f(aT)}^{h}+(bS)}^{h}}$$ (3)

where *f*(∙) are increasing Hill functions:

$${f(bV)=m}_{1}\frac{{\left(bV\right)}^{h}}{e^{h}+{\left(bV\right)}^{h}};\;{f(bV+bS)=m}_{2}\frac{{\left(bV+bS\right)}^{h}}{e^{h}+{\left(bV+bS\right)}^{h}};\;{f(aT)=e}_{0}+m_{2}\frac{(a{T)}^{h}}{e^{h}+{\left(aT\right)}^{h}};$$

*g*(∙) is a decreasing Hill function:

$$g(aT)=\frac{m_{1}}{1+{\left(\frac{aT}{e}\right)}^{h}};$$

where $\dot{x}$ denotes the time derivative of *x*, the variables *S*,*T*, and *V* are the mean firing rates of SSC, TRN, and VPL, respectively, the parameters *a*,*b*, and *θ* are coefficients varying in the interval (0,1), representing the efficacy of GABAergic transmission, glutamatergic transmission, and the self-limiting term, respectively.

The choice of using Hill functions in our thalamocortical model is suggested by literature data about neuron input-output relationships [28]. The values of the Hill function parameters have been previously set on the basis of literature data: time constant *τ* = 0.5 s; maximum firing rate of excitatory fibers *m*_1_ = 100 Hz; maximum firing rate of inhibitory fibers *m*_2_ = 80 Hz; Hill function’s half-saturation constant *e* = 20 Hz; basal half-saturation constant *e*_0_ = 20 Hz; and Hill coefficient *h* = 2.5 [18]. Having established these points, Eq (1) represents the excitatory effect of VPL glutamatergic fibers acting on SSC; Eq (2) the combined excitatory effect of VPL and SSC glutamatergic fibers acting on TRN; and Eq (3) the combination of the excitatory effect exerted by SSC glutamatergic fibers and of the inhibitory effect exerted by TRN GABAergic fibers, both acting on VPL. In Eq (3), the combined excitatory and inhibitory effects have been represented by using an increasing Hill function of variable *S* in which *m* and *e* are represented by decreasing and increasing Hill functions of variable *T*, respectively. We have adopted this kind of function because the inhibitory effect of GABAergic fibers on the response of neurons to excitatory fibers causes either a reduction of maximum firing rate (reduction of *m*), or a decrease of neuron sensitivity to excitatory pulses (increase of *e*), in both cases fitting Hill functions [28,29].

Literature data about the effects of neurosteroids on neurotransmission, and about the reciprocal interactions between endocrine axes, led us to adopt Hill functions as a suitable mathematical model also for the other functional interactions considered in this study (see below).

Relationships between the strength of the interactions and the system bifurcation point. In our previous study, we have shown that for *b* = 1, i.e. a theoretical maximal value for the glutamatergic strength, the system is monostable for *a*>0.265, with a single basin of attraction and a low-firing-rate steady state (see Box 1). At *a* = 0.265 there is a bifurcation point (see Box 1), so that if *a* goes below this value the system becomes bistable, acquiring an additional high-firing-rate steady state. In general, in the dynamics of a loop system representing a living system, the appearance of a bifurcation corresponds to a critical physiological event, as it enables a stable change of functional regime. In this case, the bifurcation enables the existence of two alternative stable steady states, involving all the three elements, or regions, of the thalamocortical loop. One steady state is characterized by basal, i.e. low-firing-rate activity, and the other one by high-firing-rate activity. The basin of attraction of the high-firing-rate steady state rapidly enlarges for decreasing *a*, making the system progressively prone to fall on the high-firing-rate steady state, which is assumed to represent the FM chronic pain condition [18].

**Fig 2:**
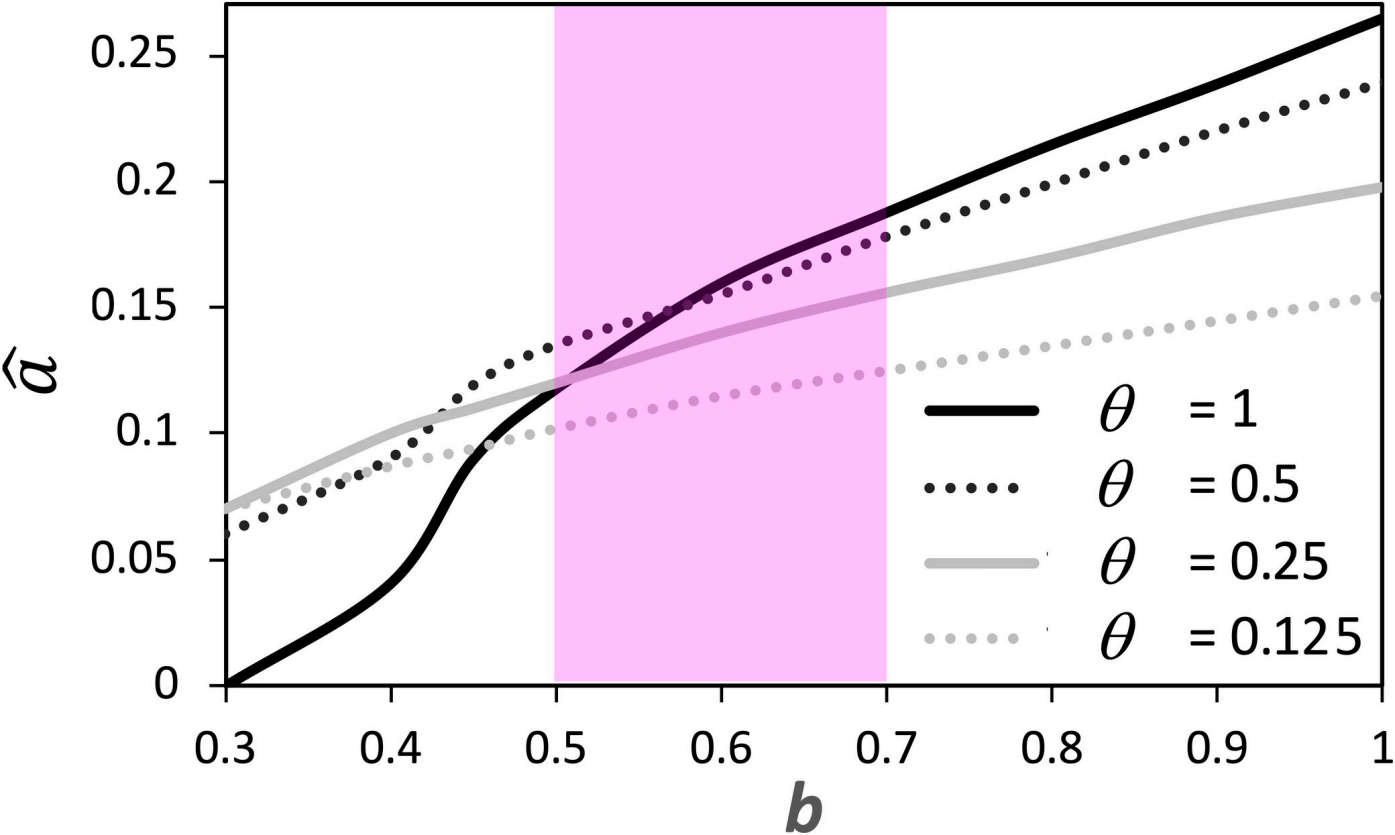
Influence on the thalamocortical system dynamics exerted by the coefficients of the differential equation variables. The coefficient *b* shows a positive, non-linear correlation with $\hat{a}$, the bifurcation value of *a*. The coefficient *θ* influences the steepness of the correlation curve. The pink area highlights a range of *b* values within which the variation of *θ* has scarce influence on the corresponding values of $\hat{a}$.

We conducted here a further analysis by considering the relationship between *b* and the bifurcation value of *a* (see Box 1), indicated as $\hat{a}$. We showed that, as *b* decreases, the value of $\hat{a}$ also decreases according to a sigmoid curve (Fig 2). This trend is conserved for different values of *θ*, but the steepness of the curve is lower for decreasing *θ* (Fig 2). The $b/\hat{a}$ curves, calculated for *θ* = 1 and *θ* = 0.5, cross each other in correspondence of *b*≌0.55, showing that for values of *b* around this point, the variation of *θ* has a limited influence on the value taken by $\hat{a}$ (Fig 2).

### New extended model: Involvement of endocrine axes in FM pathogenesis

The thalamocortical loop model explains the possible mechanism leading to chronic pain, but it does not account for the causes of the weakening of GABAergic transmission and/or glutamatergic strengthening. However, as stated above, evidence argues for the involvement in FM of the HPG and HPA axes [30]. This is consistent with our thalamocortical model since these axes are known to affect GABAergic and glutamatergic transmissions in the brain. The HPA axis consists of sequential interactions among the hypothalamus, the anterior pituitary, and the adrenal glands. It regulates responses to various stress conditions through the release into the plasma of corticosteroid hormones. This function promotes adaptation to environmental perturbations (acute stress) but can also produce detrimental changes in response to persistent traumatic agents (chronic stress) [31]. The HPG axis consists of sequential interactions among the hypothalamus, the anterior pituitary, and the gonads. It regulates reproductive activities and functions in both female and male individuals through the release into the plasma of gonadal steroids [32].

#### Influence of the HPG axis on the thalamocortical loop

Various data point to modulatory effects of gonadal hormones or their derivatives on GABAergic and glutamatergic transmissions. In female FM patients, changes in progesterone and testosterone during the menstrual cycle have shown an inverse correlation with pain intensity [33]. In the rat cerebral cortex, testosterone has been shown to upregulate the expression of the GABA_A_ receptor α2 subunit, involving higher ion currents [34]. In depressed women, anxiolytic and antidepressant effects of testosterone have been related to increased GABA levels in the posterior-cingulate cortex [35].

Neurosteroids are more directly involved in the regulation of brain neurotransmission [36]. Allopregnanolone (3α,5α-tetrahydroprogesterone), a derivative of progesterone, and its structurally related androstanediol (5α-androstane-3α,17β-diol), a derivative of testosterone, potentiate GABAergic transmission through a positive allosteric modulation of the GABA_A_ receptor [37]. They have been found to induce sedative, anxiolytic, and antiepileptic effects [38,39]. The presence of neurosteroids in the brain can derive from de novo synthesis or from the metabolism of progesterone and testosterone that cross the blood brain barrier [40,41]. Their brain levels are correlated with gonadal hormone blood levels, especially for wide fluctuations of these latter.

**Fig 3:**
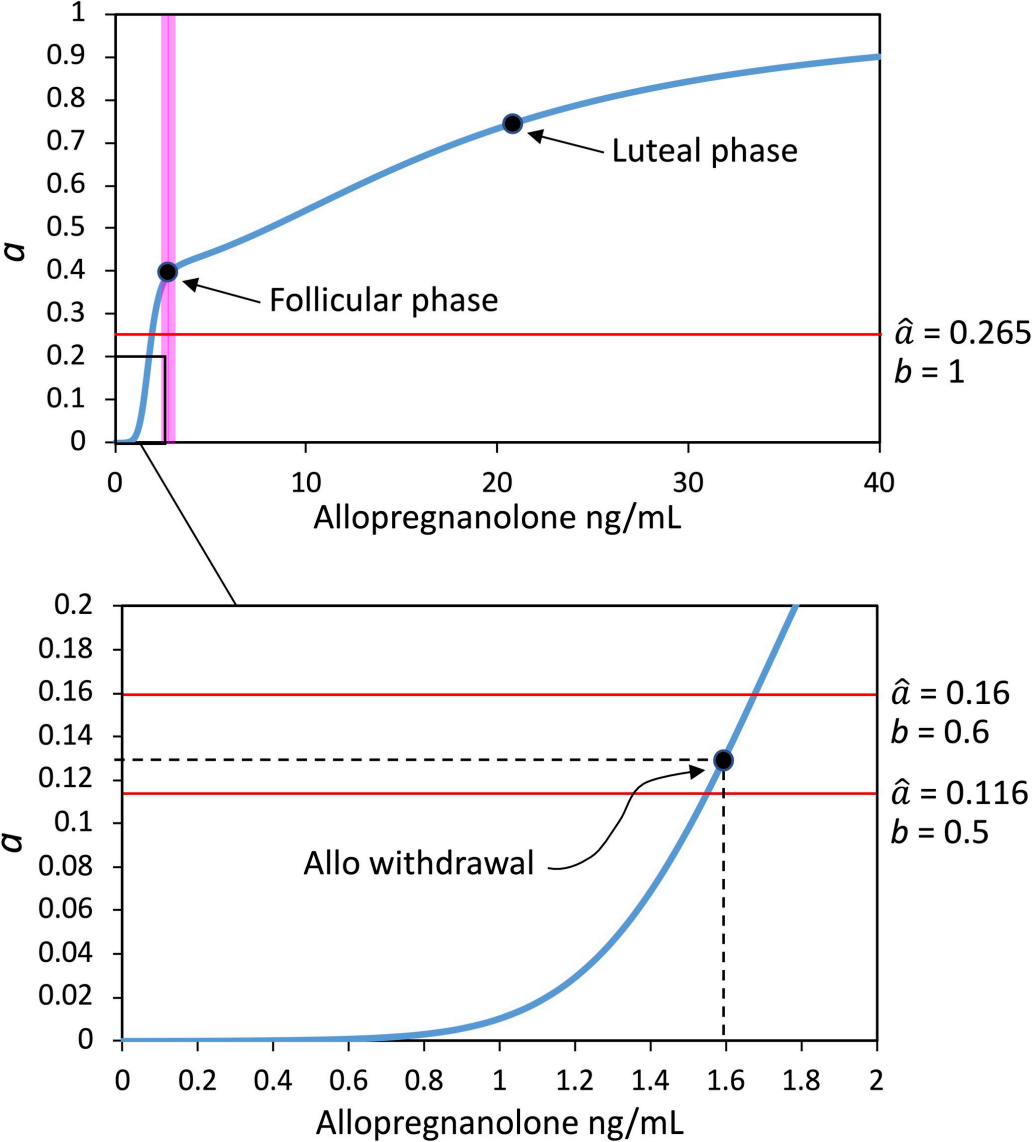
Relationship between estimated allopregnanolone brain levels and the strength of GABAergic transmission, expressed by the coefficient *a*, as derived from Eq (4). Top panel. The pink band indicates the transition zone separating two ranges of allopregnanolone where different effects on the GABA_A_ receptor are prevalent: In the lower range the effect on GABA_A_ α4 subunit expression, while in the higher range the positive allosteric modulation on GABA_A_ activity. The points corresponding to allopregnanolone concentrations and *a* values at the follicular and luteal phases, and the value of the bifurcation point at the maximal glutamatergic strength ($\hat{a}=0.265,b=1$), are indicated. Bottom panel. Zoom-in view of the bottom left inset in the top graph. The point corresponding to allopregnanolone withdrawal and related *a* value is indicated. The horizontal red lines indicate the values taken by the bifurcation point at half-maximal glutamatergic strength ($\hat{a}=0.116,b=0.5$), and for a 20% rise of *b* above this level ($\hat{a}=0.16,b=0.6$). At allopregnanolone withdrawal, the thalamocortical loop system is still monostable for *b* = 0.5 $\left(a>\hat{a}\right)$, but it becomes bistable for *b* = 0.6 $\left(a<\hat{a}\right)$.

We first considered the possible effects of allopregnanolone, given that female progesterone undergoes much wider fluctuations than male testosterone along the reproductive cycle. Allopregnanolone and other neurosteroids act on both synaptic (γ-type) and extrasynaptic (δ-type) GABA_A_ receptors, thereby prolonging phasic and enhancing tonic GABAergic inhibition, respectively [42]. By considering the total effect of neurosteroids on GABAergic transmission, we derived a quantitative relationship between allopregnanolone brain levels and the coefficient *a* in the differential equations of the thalamocortical model. To this aim, we first derived allopregnanolone plasma and brain levels at different woman reproductive phases from literature data or estimated them from the brain/plasma ratio at the luteal phase (Table 1). These are representative values, since there is a range of concentrations to be found in different phases and among different individuals, and are used to quantify the strength of GABAergic transmission, expressed in our mathematical model by parameter *a* (see also Fig 3). The model is valid regardless of the precise parameter values, and our approach and our analysis can still be adopted.

**Table 1:** Representative steroid concentrations in the woman at different reproductive phases.

Reproductive phase	Steroid	Tissue	Concentration (ng/mL)	Source
Follicular	Progesterone	Plasma	1.5	[43]
		Brain	–	–
			
	Allopregnanolone	Plasma	0.15	[43]
		Brain	2.4	Estimated*
Luteal	Progesterone	Plasma	10	[43]
		Brain	41	[43]
			
	Allopregnanolone	Plasma	1.2	[43]
		Brain	20	[43]
Late pregnancy	Progesterone	Plasma	204	[44]
		Brain	–	–
			
	Allopregnanolone	Plasma	16	[44]
		Brain	260	Estimated*

* = estimated from the brain/plasma ratio at the luteal phase.

We then derived from the literature the parameters of dose-response curves for the effect of allopregnanolone on GABA_A_ receptors. These curves fit Hill functions, and therefore, we obtained median values for Hill curve parameters (Table 2). In a couple of studies, values of baseline GABA_A_ activities were also indicated, allowing us to estimate the maximum increase of receptor activity induced by allopregnanolone as 100% and 200% with respect to baseline, respectively [45,46].

**Table 2:** Hill function parameters derived from the curve fitting of allopregnanolone effects on GABA_A_ receptors.

Experimental model	n	*h* ^a^	*e* ^b^ (ng/mL)	*e* ^c^ (%)	Threshold dose for max effect (ng/mL)	Ref.
Bovine chromaffin cells	5	2	15	20	–	[47]
Rat hippocampus/cortex	6	2	45	8	600	[45]
*Xenopus* oocytes	6	2	300	2	1500	[48]
Rat neurons	5	2	18	5	150	[49]
Mouse L-tk cells	8	1.2	9	0.3	300	[50]
Rat dentate granule cells	7	1.15	3.9	5	90	[46]
Rat embryo hippocampal neurons	7	2.8	300	4.8	900	[51]
Median values		2	18	5	450	

^a^Hill coefficient, ^b^Hill function’s half-saturation constant derived from raw data, ^c^Hill function’s half-saturation constant derived from data standardized on a 0–100 scale. The half-saturation constant corresponds to the EC_50_, i.e. the input concentration inducing 50% output response.

Hence, the allopregnanolone modulation of GABA_A_ activity at different female reproductive phases was quantified as follows. The value *a* = 1 was set as the maximum reachable GABA_A_ activity, i.e. the activity observed at the highest pharmacological doses of allopregnanolone, ranging in the interval 90–1500 ng/mL, with a median of 450 ng/mL (Table 2). Conversely, the minimum physiological effect of allopregnanolone was assumed to occur at follicular brain levels, estimated at 2.4 ng/mL (Table 1). At the follicular phase, the value of *a* was assumed to be proximal to the basal GABA_A_ activity observed in the absence of allopregnanolone in experimental studies. Therefore, by considering an average maximum increase of 150%, the value of *a* at the follicular phase (*a*_*f*_) was derived from the equation *a*_*f*_+1.5*a*_*f*_ = 1, yielding *a*_*f*_ = 0.4. Consequently, the EC_50_ of allopregnanolone, taken as the median value of the half-saturation constant in Table 2, 18 ng/mL, should correspond to *a* = 0.7, i.e. the midpoint between 0.4 and 1. Based on these estimates, the value of *a* = 1 is never reached around the female cycle, since luteal allopregnanolone brain levels are around 20 ng/mL, i.e. definitely below the doses inducing maximum GABA_A_ activation in experimental studies (Table 2).

The values of *a* corresponding to allopregnanolone brain levels ranging from the follicular to the luteal phase, are always above the bifurcation point of our thalamocortical loop systems. As previously shown, the bifurcation value is $\hat{a}=0.265$ for a maximum value of *b* = 1, while it even scales down for lower *b* values. Hence, we must argue whether GABAergic strength below the bifurcation level can occur in the organism. A positive answer seems to come from a depressive effect of very low allopregnanolone levels on GABA_A_ activity, driving GABAergic strength below basal follicular levels. Perimenstrual or post-partum progesterone drop has been reported to cause brain allopregnanolone withdrawal. Due to its positive effect on GABA_A_, allopregnanolone has sedative, anxiolytic, and anticonvulsant properties, and accordingly, allopregnanolone withdrawal has been correlated to premenstrual syndromes, such as anxiety and seizure susceptibility [52–54].

In a progesterone-withdrawal paradigm in the rat, mimicking post-menstrual and post-partum syndromes, reduced levels of allopregnanolone enhance the expression of GABA_A_ α4 subunit, involving GABA_A_ reduced activity [55]. Such an effect is distinct from the positive allosteric modulation of GABA_A_ at higher allopregnanolone levels, and it has been shown to depend on the upregulation of the early growth response factor-3 (Egr3) [56]. This effect involves a 6-fold decrease in the GABA_A_ current time constant. Hence, by considering the ion current decay equation:

$$I=I_{0}e^{–\frac{t}{\tau }},$$

where *I* is the current at time *t*,*I*_0_ is the current at time *t* = 0 (set to *I*_0_ = 1),*τ* is the time constant, and integrating for *τ* = 1 and *τ* = 1/6 as follows:

$$I_{TOT}=I_{0}\int_{0}^{\infty }e^{–\frac{t}{\tau }}dt,$$

an about 3-fold decrease of GABA_A_ currents was inferred. Hence, by applying a 3-fold decrease to the previously derived follicular value of *a* = 0.4, a value of *a*≌0.13 was inferred for allopregnanolone withdrawal.

In addition, to derive an estimate of allopregnanolone brain concentrations at withdrawal, we referred to an animal model of perimenstrual catamenial epilepsy in which allopregnanolone withdrawal has been obtained through the administration of the 5α-reductase inhibitor finasteride [57]. Finasteride has induced in female rats a reduction of allopregnanolone plasma levels from 9 to 6 ng/mL. Hence, assuming a similar reduction for allopregnanolone brain levels in women, by starting from the above estimated brain follicular level of 2.4 ng/mL, we can roughly estimate a withdrawal allopregnanolone brain level of 1.6 ng/mL, to be put in correspondence with the above-derived value of *a* = 0.13.

To sum up, we modeled the effect of allopregnanolone on the strength of GABAergic transmission by considering two independent actions. One occurs starting from follicular to luteal and pregnancy brain levels, with a dose-dependent rise of GABAergic strength due to positive GABA_A_ modulation. The other one occurs for allopregnanolone sub-follicular, withdrawal levels, pulling down GABAergic strength due to a rearrangement of GABA_A_ α subunits. The mathematical expression for such a combined effect is the sum of two increasing Hill functions:

$$a=f_{w}(A)+f_{g}(A),$$ (4)

where *f*_*w*_(*A*) is the withdrawal component:

$$f_{w}(A)=a_{f}\frac{A^{h_{w}}}{\left(e_{w}^{h_{w}}+A^{h_{w}}\right)},$$ (5)

*f*_*g*_(*A*) is the positive allosteric component:

$$f_{g}(A)=(1-a_{f})\frac{A^{h_{g}}}{\left(e_{g}^{h_{g}}+A^{h_{g}}\right)},$$ (6)

*A* is the brain allopregnanolone concentration, *a* is the GABAergic strength, *a*_*f*_ is the value of *a* at the follicular *A* level, *e*_*w*_ and *h*_*w*_ are the EC_50_ and Hill coefficient for the genomic effect induced by allopregnanolone on the GABA_A_ α subunit at withdrawal brain concentrations, and *e*_*g*_ and *h*_*g*_ are the EC_50_ and Hill coefficient for the positive allosteric effect on GABA_A_. For computational analysis, we adopted *e*_*g*_ and *h*_*g*_ values corresponding to the median values reported in Table 2. Conversely, no literature data are available for *e*_*w*_ and *h*_*w*_, except that, in general, steroid hormone genomic effects fit a Hill function (see below). Therefore, given that the withdrawal effect occurs at sub-follicular allopregnanolone levels, we considered a Hill function having maximum at the follicular value of *a*_*f*_ = 0.4, asymptotically approached at a value of *A*≌2.4, and admitting a solution for the above-reported values, *A* = 1.6 and *a* = 0.13. The derived values for the parameters of the Hill functions in Eqs (5) and (6) are reported in Table 3.

**Table 3:** Hill function parameters used in Eqs (4), (5) and (6).

Allopregnanolone effect	Allopregnanolone range	Hill function	*e*	*h*	*a*_*f*_
GABA_A_ activity	Follicular-luteal-pregnancy	*f*_*g*_(*A*)	18	2	0.4
α4 subunit expression	Withdrawal-luteal	*f*_*w*_(*A*)	1.8	6.5	0.4

Symbols as in Table 2. The values of *e* and *a*_*f*_ are expressed as ng/mL

Eq (4) describes the predominance of the withdrawal effect of allopregnanolone on the strength of GABAergic transmission for subfollicular brain levels, i.e. concentrations ranging between 0 and 2.4 ng/mL, while the positive allosteric GABA_A_ modulation is predominant for concentrations above 2.4 ng/mL (Fig 3). As shown in the figure, allopregnanolone withdrawal drives the coefficient *a* down to an estimated value of 0.13. To understand possible pathogenic consequences of such a decrease, this value must be related on the one hand to the bifurcation value $\hat{a}$, and on the other hand to the strength of glutamatergic transmission *b*, as determined by the dynamics of our thalamocortical model.

#### Influence of the HPA axis on the thalamocortical loop

Indirect data on N-methyl-D-aspartate receptor (NMDAR)-dependent cytosolic Ca^2+^ rise suggest that glutamate-induced excitotoxicity involves an about 100% increase of glutamatergic transmission [58], which in our model can be assumed as maximum glutamatergic strength, corresponding to *b* = 1. Hence, we can estimate that a value of *b* = 0.5 represents physiological glutamatergic strength, because a 100% increase of *b* = 0.5 yields *b* = 1. According to the $b/\hat{a}$ relationship depicted in Fig 2, *b* = 0.5 corresponds to $\hat{a}=0.116$, i.e. a bifurcation point that is close to, but still below, the value of *a* = 0.13 estimated for allopregnanolone withdrawal. However, if *b* reaches a value of 0.6, the bifurcation value becomes $\hat{a}=0.16$. Hence, a 20% increase of glutamatergic strength would be sufficient to drive the bifurcation point above the reduction of GABAergic strength imposed by allopregnanolone withdrawal (Fig 3). It should be noted that the numerical values of our analysis have been estimated from indirect data; therefore, they are not expected to exactly measure real transition processes leading to FM. Yet, our model allows us to conclude that a strong reduction of GABAergic transmission combined with a mild rise of glutamatergic transmission are likely to induce a pathogenic functional state in the thalamocortical loop system.

We then explored possible causes leading to an increase of glutamatergic transmission able to induce an FM pathogenic condition. Consistent with the correlation between FM and acute or chronic stress, the activation of the HPA axis is known to induce glutamatergic strengthening [59–61]. This relationship is difficult to estimate quantitatively, since various corticosteroids are released upon HPA axis activation, inducing different effects on central neurotransmission. For instance, pituitary ACTH stimulates adrenals to release allopregnanolone and THDOC, both passing the brain barrier and acting as positive allosteric modulators (PAM) of GABA_A_ receptors, being part of a homeostatic mechanism that limit the impact of HPA axis activation [62]. Cortisol is also known to induce negative feedback on the HPA axis by suppressing the release of glutamate and facilitating that of GABA in magnocellular neurons of the hypothalamic supraoptic and paraventricular nuclei [63]. Conversely, dehydroepiandrosterone (DHEA) and its sulphated derivative DHEA-S, also released by adrenals, act as negative modulators of GABA_A_ and positive modulators of NMDA glutamate receptors [64]. In addition, cortisol has also been shown to act pre- and post-synaptically by facilitating glutamatergic transmission [59]. This action occurs in various brain areas, including the hippocampus, amygdala, prefrontal and frontal cortex [60,61,65]. Accordingly, the first global effect of acute stress is a rapid decrease of GABAergic transmission together with an increase of glutamate transmission in brain areas involved in cognitive functions, such as prefrontal cortex and hippocampus [66,67].

Hence, a central GABA/glutamate unbalance in favor of brain network excitability can be considered a distinctive consequence of HPA axis activation. Such an effect can also be exacerbated under specific conditions. A mental stressor test on humans showed that the homeostatic allopregnanolone response to HPA axis activation was absent in postpartum women [68], and was negatively correlated with baseline allopregnanolone levels in men [69], thus possibly enhancing the GABA/glutamate unbalance.

### HPG and HPA axes as a loop system

#### Double-inhibitory interaction between the HPG and HPA axes

Until now, we have considered HPG and HPA axes as independent units inducing distinct effects on neurotransmission. However, evidence argues for the possibility that, under some circumstances, these axes become entangled and form a feedback loop due to a reciprocal inhibitory action [70]. Available data indicate that weak correlations are observed for circadian or follicular-to-luteal fluctuations of these axes, while significant negative correlations are found for wider fluctuations under stress conditions, perimenstrual, pregnancy, or pharmacological treatments [71].

Repression of the HPA axis is due to inhibitory activities exerted by estrogens, androgens, progesterone, and their metabolites on neurons of the hypothalamic peri-paraventricular and paraventricular nuclei, and the anterior pituitary. Repression of the HPG axis is due to inhibitory activities exerted by corticosteroids on hypothalamic kisspeptin and gonadotropin-releasing-hormone (GnRH) neurons, the anterior pituitary, and gonads [72].

Variable or absent correlations between the circadian profiles of progesterone and cortisol have been reported for cycling and postmenopausal women [73,74]. Conversely, various studies have reported evidence of HPG-HPA interactions for wider hormone fluctuations. In gilt pigs, treatments inducing sustained elevation of cortisol, but not acute elevation, have inhibited the luteinizing hormone (LH) surge, together with estrus and ovulation [75]. In female monkeys, elevation of serum cortisol by tissue infusion has decreased the availability of progesterone to target organs [76]. In pregnant women, the circadian variation of progesterone has been found inversely correlated with that of cortisol [77,78], while intravenous cortisol infusion has caused transient estrogen, progesterone, and androgen suppression [79]. Hydrocortisone administration to eumenorrheic women in the early follicular phase has slowed down hypothalamic gonadotropin-releasing hormone (GnRH) and pituitary LH pulse frequency [80]. A study on premenstrual dysphoric disorder (PMDD) has reported higher levels of allopregnanolone and lower nocturnal cortisol levels in patients, compared with healthy controls [81]. An in vitro study on primary cultures of human placenta has indicated cortisol as an antiprogestin causing pre-labor progesterone withdrawal in pregnant women [82]. HPG-HPA interference can also occur via an indirect mechanism, since enhanced synthesis of cortisol may reduce the amount of pregnenolone available for the synthesis of progesterone [83].

HPG-HPA interactions have also been highlighted in mammal males. In bulls and rams, administration of the synthetic glucocorticoid dexamethasone has induced lowering effects on circulating LH and testosterone by acting at the hypothalamic level [84,85]. In human adult males, pharmacologically increased cortisol plasma level has shown a detrimental effect on circulating testosterone [86]. Similarly, endurance exercise has caused cortisol plasma level increase concurrently with testosterone decrease [87]. On the other side, testosterone replacement has lowered cortisol levels and cortisol excursion after corticotropin-releasing hormone (CRH) stimulation tests [88].

#### Possible molecular mechanism of the HPG-HPA double-inhibitory interaction

Different studies have provided insight about possible mechanisms explaining the inverse correlation between HPG and HPA axes. In vertebrate models, stress-induced glucocorticoids repress the HPG axis by inhibiting GnRH neurons or stimulating neurons releasing gonadotropin-inhibitory hormone (GnIH) [89–91]. In castrated male rats, electroshock-induced stress has inhibited LH release by acting through CRH release [92]. Allopregnanolone has induced the GABAergic inhibition of HPA axis activation in female rats [93,94] and pregnant women [95].

However, the data that explains more clearly the double negative HPG-HPA interaction for wide hormone fluctuations are those that point to the role of GABA and glutamate. In rodents and humans, pituitary LH secretion is stimulated by GnRH neurons that are prevalently activated by the neurotransmitter kisspeptin, released by two other groups of neurons, one located rostrally in the preoptic area (POA), and the other more caudally in the arcuate nucleus (ARC) [96,97]. The ARC kisspeptin neurons receive negative estradiol feedback and induce small, pulsatile GnRH and LH release. The POA kisspeptin neurons receive positive estradiol feedback and are responsible for the large GnRH and LH surge preceding ovulation [98]. Both neuron populations are sexually dimorphic in sheep and humans. Male rodents have lower kisspeptin neurons in the rostral periventricular area of the third ventricle, possibly explaining male inability to mount the estrogen-induced LH surge [99].

Even though estradiol is considered the main regulator of gonadotropin release, GnRH and kisspeptin neurons express various glutamate and GABA receptors and respond to these neurotransmitters [100,101]. Different data converge to indicate that the LH surge is stimulated by glutamate [102,103] and inhibited by GABA [104,105], and that its occurrence is coincident with a decline of GABAergic transmission to both POA kisspeptin neurons and GnRH neurons [106,107].

On the HPA axis side, the CRH neurons in the hypothalamic paraventricular nucleus require a tight regulation predominantly exerted by three neurotransmitters: GABA, glutamate, and norepinephrine [108–110]. Glutamate is excitatory on CRH neurons, whereas GABA exerts both phasic and tonic inhibition, mediated by synaptic and extrasynaptic GABA_A_ receptors, respectively [111]. Noradrenergic inputs originating in the brainstem act on peri- and intra-paraventricular GABAergic and glutamatergic interneurons to regulate CRH release. Norepinephrine both suppresses and enhances GABAergic inhibition, whereas its excitatory effect on the HPA axis is mainly due to a facilitation of glutamate release onto CRH neurons [110].

Brain regions implicated in the integration of psychogenic and social stressor stimuli, such as the hippocampus, amygdala, and prefrontal cortex, regulate CRH neurons activity through complex polysynaptic routes with multiple relays. These pathways include the bed nucleus of the stria terminalis, the medial preoptic area, and peri-paraventricular neurons, among others. The vast majority of these routes converge onto the regulation of the GABAergic transmission to the CRH neurons, that is stimulated by inputs from the hippocampus and the prefrontal cortex (downregulating the stress response), and inhibited by the activation of the amygdala (stimulating the stress response) [112]. However, the GABAergic synapse shows different forms of plasticity following acute and chronic stress. For example, immediately after the onset of stress the GABA synapse on CRH neurons becomes excitatory, due to the disruption of the chloride gradient induced by norepinephrine action. This effect is necessary for the attenuation of the inhibiting GABA action and the disinhibition of the HPA axis [113].

#### Quantitative evidence of the double-inhibitory interaction

To obtain a mathematical model of the interplay between HPG and HPA axes, we started from an analysis of the correlation between the plasma levels of glucocorticoids and gonadal hormones such as progesterone and testosterone, which, as we have seen, induce directly or indirectly the strongest effects on GABAergic and glutamatergic transmission. We considered a series of studies on both animals and humans reporting combined measurements of corticosteroids and gonadal hormones. As stated above, we found weak correlation or partial positive correlation for hormone circadian oscillations; see e.g. [114–116]. By contrast, significant negative correlations were found in studies reporting the following data: (i) plasma values of glucocorticoids induced by stress condition, together with the reciprocal variations of gonadal hormones; (ii) plasma values of gonadal hormones caused by pregnancy or pharmacological treatment, together with the reciprocal variations of glucocorticoids (Table 4). Dose-response relationships were derived by considering the hormone whose variation was induced by environmental, reproductive, or pharmacological effects, as the input (independent) variable, and the other hormone as the output (dependent) variable. In a couple of cases these roles were undefined and two dose-response curves were obtained by flipping the two variables with each other. A decreasing Hill function was fitted to data, whose function parameters were derived after data normalization on a 0–100 scale. (Table 4).

**Table 4:** Hill function parameters determined by fitting to matched samples of gonadal hormone/corticosteroid plasma levels.

*x* ^a^	*y* ^b^	*e* ^c^	*h* ^d^	*n* ^e^	*p* ^f^	Species	Ref.
corticosterone	progesterone	1	4.2	6	< 0.001	chicken	[117]
corticosterone	testosterone	5	3.9	4	< 0.001	chicken	[117]
cortisol	testosterone	28	5.2	6	< 0.001	rabbit	[118]
cortisol	LH	28	2.4	13	< 0.001	swine	[75]
cortisol	progesterone	23	8	8	< 0.001	humans	[119]
cortisol	testosterone	22	6	10	< 0.001	men	[87]
cortisol	testosterone	48	2.4	71	< 0.001	men	[86]
progesterone	corticosterone	32	3.5	11	< 0.001	chicken	[120]
testosterone	cortisol	17	7.3	6	< 0.001	rabbit	[118]
testosterone	cortisol	58	7.5	12	< 0.001	rabbit	[118]
LH	cortisol	50	7.5	6	< 0.001	rabbit	[118]
LH	cortisol	51	4.1	12	< 0.001	rabbit	[118]
testosterone	cortisol	23	1.5	71	< 0.001	male humans	[86]
progesterone	cortisol	64	3.8	23	< 0.001	female humans	[77]
progesterone	cortisol	65	8	6	< 0.001	female humans	[121]
		28^‡^	4.2^‡^				

^a^Input variable, expressed as hormone plasma level standardized on a 0–100 scale

^b^output variable, expressed as the input variable

^c^Hill function’s half-saturation constant

^d^Hill coefficient

^e^size of the matched sample

^f^correlation p-value

^‡^median values.

The median value of the Hill coefficient for endocrine axis interactions, *h* = 4.2 (Table 4), and the one for the genomic allopregnanolone effect on GABA_A_, *h* = 6.5 (Table 3), are higher than those derived for the input-output neuron firing rate relationship of our thalamocortical model, *h* = 2.5 [18], and for the allopregnanolone positive allosteric modulation on GABA_A_, *h* = 2 (Table 3). The Hill coefficient *h* is a measure of cooperativity in interacting systems [122] and hence, it is conceivable that direct effects on neuron electrophysiology yield lower values of *h* than genomic effects or interactions between endocrine axes. Therefore, we can take the different values of *h* as further evidence that the Hill function is a suitable fit for the kind of interactions that we have analyzed. This agrees with general evidence that the Hill function fits stimulus-response data in a wide set of biological systems [123–128].

#### Dynamic model of the HPG-HPA interaction

After having acquired data about the mathematical expression of the mutual HPG-HPA interaction, and having estimated the values of its parameters, we modelled the overall interaction as a dynamic system (see Box 1). As shown above, the hypothalamic neurons responsible for the activation of the HPA and HPG axes receive inputs from various brain pathways, which in turn are influenced by HPA and HPG hormones or their brain derivatives. The realization of an extensive computational model for this network of positive and negative feedback interactions is currently impossible due to the lack of suitable qualitative and quantitative data. However, two macroscopic effects can be considered: (i) the existence of a negative feedback mechanism (self-inhibition) in each axis; (ii) the double-negative interaction between the two axes (mutual inhibition) emerging for wide oscillations of gonadal and/or corticosteroid hormones.

**Fig 4:**
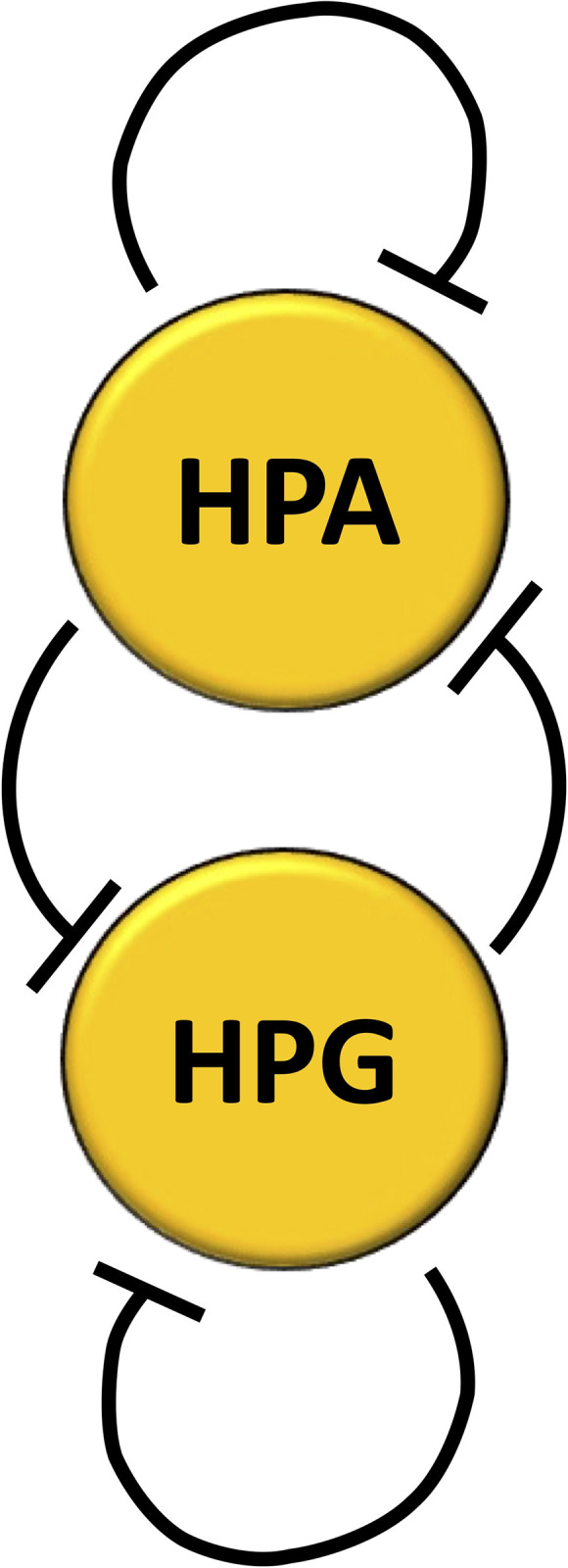
Simplified diagram of the feedback loop system formed by the HPA and HPG axes. Line ends as in Fig 1.

We therefore considered a simplified loop system consisting of two elements, each representing one of the axes. From a mathematical standpoint, each of the endocrine axes can be seen as a monotone aggregate of subsystems and therefore considered as a single, condensed element in the mathematical model of the considered dynamic system, still enabling the analysis of its behavior based on the presence of signed loops [129]. In our simplified model, two inhibitory arcs start from each node (element): one is a negative self-loop that represents the self-inhibition of the axis, while the other is directed to the other element and represents part of the double-inhibitory interplay between the two axes (Fig 4).

The dynamics of this system were mathematically described by two differential equations involving two state variables that represent the activity of the endocrine axes on a 0–100 scale (e.g. referring to the plasma concentration of cortisol for the HPA axis and of progesterone for the HPG axis). The mathematical model accounts for both the self-inhibition due to the negative feedback of each axis (self-limiting factor) and the mutual inhibition between the axes (double-inhibitory, positive loop), which becomes significant at high hormone plasma levels. The variation of the activity of each axis over time was set to depend on the activity of the other axis (mutual inhibition), according to a decreasing function, and on itself, according to a decreasing function (self-inhibition). Decreasing Hill functions, which generally provide the best fit for endocrine effects (see above), were chosen to describe all these interactions. The system of differential equations was therefore defined as follows:

$$\tau \dot{∙HPA}=f(HPG)–k(HPA)∙HPA$$ (7)

$$\tau \dot{∙HPG}=f(HPA)–k(HPG)∙HPG$$ (8)

where the variables *HPA* and *HPG* represent the activities of the axes, *τ* is the time constant (equal for both axes), and $\dot{x}$ denotes the time derivative of *x*. The two functions *f*(∙) and *k*(∙) appear in both Eqs (7) and (8), applied to two different variables. The functions *f*(∙) are decreasing Hill functions that take values in the interval (0,100] and have the form:

$$f(x)=\frac{100}{1+{\left(\frac{x}{e_{x}}\right)}^{h_{x}}}$$

where *e*_*x*_ and *h*_*x*_ are the parameters of the Hill function, as defined above (Table 5). The self-limiting functions *k*(∙) are shifted decreasing Hill functions that take values in the interval (1,5] and can be written as:

$$k(x)=1+\frac{4}{1+{\left(\frac{\alpha ∙x}{e_{k}}\right)}^{h_{k}}}$$

where parameter *α*∈(0,1) modulates the self-limiting effect, while *e*_*x*_ and *h*_*x*_ are the parameters of the Hill function, as above (Table 5). The constant offset is needed to obtain values of *k*(*x*) that asymptotically approach 1 as *x* tends to infinity (lower importance of the self-limiting factor) and approach 5 as *x* tends to zero (higher importance of the self-limiting factor). When the value of the function *k* tends to 5, the self-limiting mechanism of each axis dominates over the mutual inhibition between the axes; in the absence of mutual inhibition, the two axes would behave as two independent systems, each with its own self-inhibition. Conversely, when the value of the function *k* tends to 1, the mutual inhibition dominates; in the absence of self-inhibition, the system would behave as a double-inhibitory positive loop and thus admit two stable equilibrium points (see Box 1), one with high *HPA* and low *HPG*, and the other with low *HPA* and high *HPG*. The parameters of the differential equations were chosen identical for both axes. The time constant *τ*, representing the timescale of the response of each element, was set to 30 min, which is typical for endocrine responses. The parameters of the Hill functions describing the interactions between axes were set equal to the median values of the data reported in Table 4. The parameters of the Hill function representing the self-limiting effect were set so that the resulting system behavior is consistent with the observed patterns of correlation between HPA and HPG axes for different intensities of their activity.

**Table 5:** Values of the parameters in the differential equations describing the interactions among axes.

Interaction	Hill function	Parameter value
Double inhibition	$f(x)=\frac{100}{1+{\left(\frac{x}{e_{x}}\right)}^{h_{x}}}$	*e*_*x*_ = 28 ^a^
		*h*_*x*_ = 4.2 ^b^
Self-limiting effect	$k(x)=1+\frac{4}{1+{\left(\frac{\alpha ∙x}{e_{k}}\right)}^{h_{k}}}$	*e*_*k*_ = 25.57 ^a^
		*h*_*k*_ = 6.6 ^b^
		α = 0.48 ^b^

Units

^a^ng/mL

^b^dimensionless.

**Fig 5:**
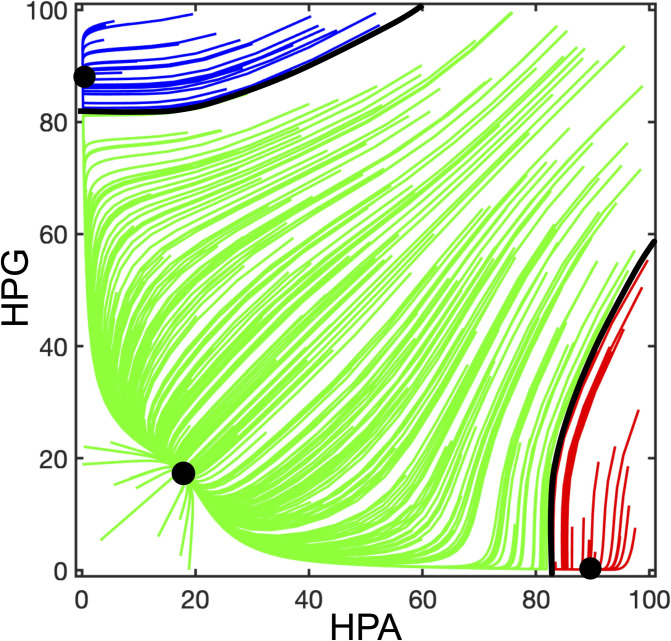
Phase portrait of the dynamic system representing the HPG-HPA interaction according to the diagram of Fig 4. The trajectories of the dynamic system have been computed with MATLAB by using Eqs (7) and (8) with the parameters reported in Table 5. Initial conditions have been randomly selected to be representative of different possible evolutions of the system. Each trajectory converges to one of the three stable equilibrium points (black dots). Black lines mark the boundaries between the basins of attraction of the three equilibria. The basin of attraction of the equilibrium point with high HPA and low HPG activity (which attracts the red trajectories) is assumed to represent the functional states leading to the onset of FM.

The numerical simulations of the complete system dynamics showed the existence of 3 stable equilibrium points, as shown in the phase portrait (see Box 1) of Fig 5. With reference to FM pathogenesis, it is interesting to analyze the behavior of the system for extreme values of *HPA* and *HPG*. When both variables are high (upper-right corner in the phase portrait), the value of function *k* tends to 1 for both axes and the system behavior is dominated by the mutual inhibition (giving rise to a positive loop) which should drive the trajectories towards either of the opposite steady states (upper-left and lower-right corners of the phase portrait). However, due to mutual inhibition, both variables initially tend to decrease and hence the value of *k* increases, thus making the self-limiting effect of each axis more and more prevalent. Due to this mechanism, the system trajectories converge to the steady state corresponding to low activity for both axes (lower-left corner of the phase portrait). Similarly, if the activity of both axes is medium or low the system is also attracted to the lower-left equilibrium point, which has the widest basin of attraction (see Box 1). It should be noted that endocrine axes never reach a constant value of activity, but rather undergo circadian fluctuations. Hence, the system should actually converge not to a fixed point, but rather to a limit cycle representing a Lissajous figure, i.e. the combination of two independent cycles with different periods, representing circadian variations of the HPG and HPA axes. However, since the range of circadian endocrine activities is confined with respect to the wide fluctuations that are expected to play a role in FM pathogenesis, they can be reasonably neglected when modeling the pathogenesis process.

On the other hand, if one variable is high and the other is low, the self-limiting effects do not prevail over the double-inhibitory mutual interaction and the system converges to either of the equilibrium points at the upper-left or lower-right corner of the phase portrait. The basin of attraction of the lower-right equilibrium represents a functional zone characterized by low HPG axis activity, e.g. the one corresponding to allopregnanolone withdrawal, and high HPA axis activity, e.g. induced by some stressful condition. Hence, such a mix of conditions drives the system towards a stable equilibrium associated with an inhibitory effect on GABAergic transmission and a stimulatory effect on glutamatergic transmission in the thalamocortical loop. This causes bistability in this brain loop network and its likely stabilization on a high-firing-rate steady state.

### Comprehensive multistable model of FM pathogenesis

#### Combined role of the endocrine and thalamocortical loops

The mutual inhibition between HPG and HPA axes gives rise to a positive loop; therefore, the dynamic system is a candidate multistable system that can admit multiple equilibria [130]. Hence, similarly to the thalamocortical loop, this endocrine loop can behave as a switch driving pathogenesis [27]. Such double-inhibitory loop operates physiological functions [72,131], but under peculiar circumstances could lead to functional disorders.

**Fig 6:**
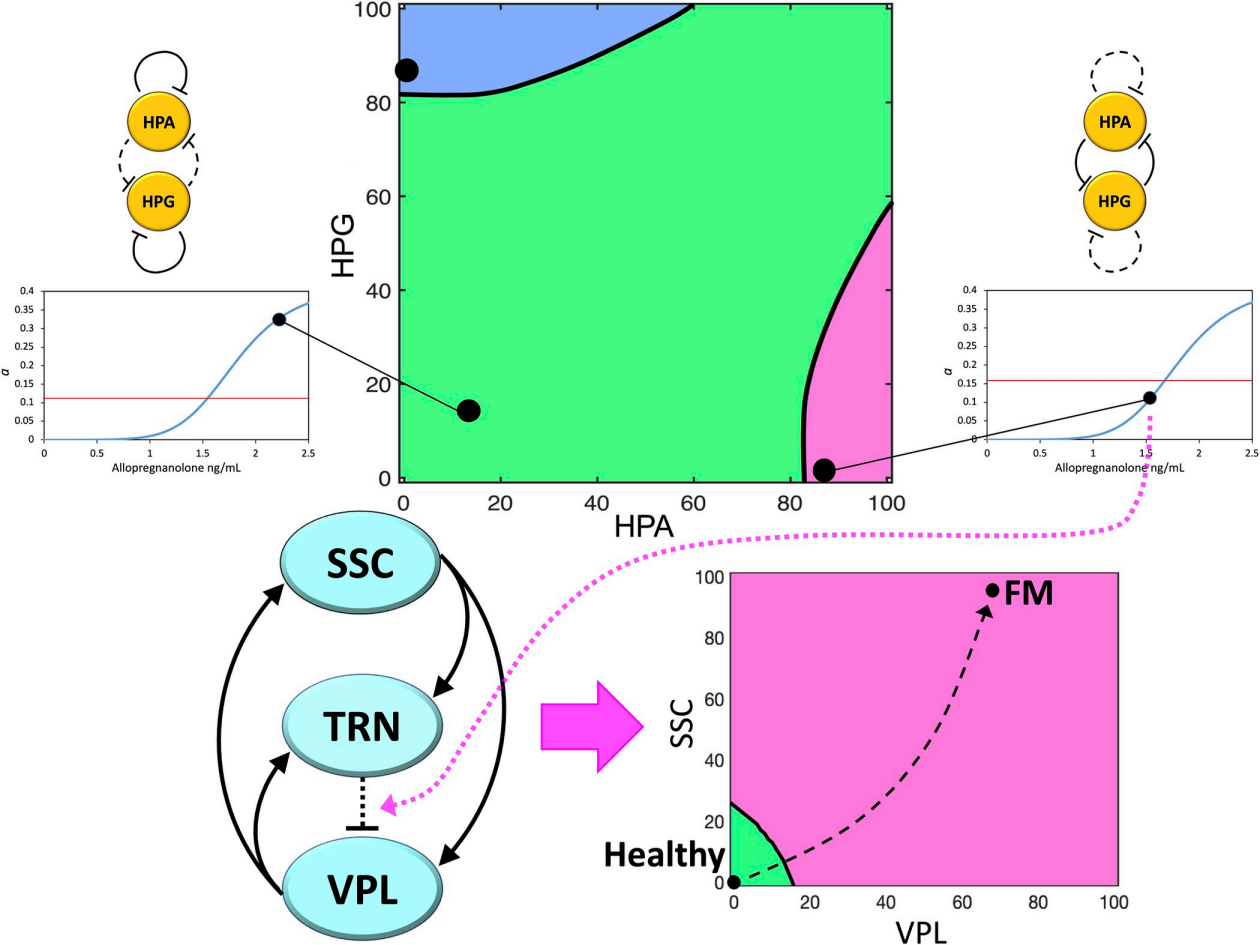
Global representation of transitions leading to FM pathogenesis according to our neuroendocrine multistable model. Top strip: Phase portrait of the HPA/HPG loop system, showing three stable equilibria (main graph). The lower-left (circadian) and lower-right (pathogenic) equilibria are connected with corresponding points on the graphs of the relationship between allopregnanolone brain concentration and GABAergic strength *a* (side graphs). The red lines in the side graphs indicate the bifurcation values $\hat{a}$, showing that the lower-right equilibrium point in the phase space leads to a value $a<\hat{a}$. Bottom strip: The pain processing thalamocortical loop (left diagram), under the influence of the lower-right equilibrium point of the endocrine loop, crosses its bifurcation point, which leads to the appearance of two stable equilibria in its own phase space (right diagram). The high-firing-rate equilibrium represents the chronic pain condition and has a much wider basin of attraction. Therefore, once bistability has arisen, the thalamocortical system is prone to converging to the high-firing-rate equilibrium, thus causing the onset of FM symptoms. The variables considered in the endocrine phase space (top) represent the percent activities of the endocrine axes, e.g. estimated from the plasma levels of cortisol (HPA) and progesterone (HPG). The phase space shown in the bottom strip is a bidimensional projection on the *VPL*/*SSC* plane of the three-dimensional thalamocortical phase space, and the graph axes represent the percent firing rate above baseline of the thalamic ventroposterolateral nucleus (VPL) and the primary somatosensory cortex (SSC).

As we have seen, a wide complex of data suggest that the conditions leading HPG and HPA axes to develop a prevalent double-inhibitory loop are the same that induce a pathogenic bistability on the thalamocortical loop. A switch of the endocrine loop to the high-HPA/low-HPG steady state could produce sustained weakening of GABAergic and strengthening of glutamatergic transmissions in the pain processing thalamocortical loop. This would considerably increase the probability of the thalamocortical loop to cross the bifurcation point, thus causing its transition to bistability, with an immediate tendency to fall on a hyperexcitation steady state leading to the development of FM (Fig 6).

In conclusion, our model, consisting of two interconnected dynamic systems, provides a theoretical explanation of a transition leading to FM pathogenesis starting from possible primary causes acting on endocrine mechanisms. The high-firing-rate activity of the thalamocortical loop is assumed to produce anomalous pain processing leading to chronic pain. This could induce cascade events in other brain regions causing the onset of symptoms that accompany pain in the FM syndrome. Alternatively, these symptoms could derive from the parallel occurrence of GABA/glutamate unbalance in other brain networks arranged as the thalamocortical loop.

#### Predictivity and limitation of the model

Our model predicts that a combination of high-HPA/low-HPG activities is a predisposing condition for FM. Hence, it provides a unifying explanation for the supposed multifactorial origin of the disease [1], by making a variety of triggering events upstream HPA and HPG axes to converge to a common neuroendocrine mechanism. However, it does not make predictions about the frequency of such occurrences in the population. For implementing this prediction, further modeling should be developed based on statistical data, including, e.g. for women, the frequency of excessively high cholesterol, the occurrence of one allopregnanolone withdrawal per cycle or pregnancy, and the individual variability of cholesterol and allopregnanolone levels during these episodes, which is beyond the aim of this study. However, the model can be tested for FM gender prevalence. By assuming a total of about 460 cycles for each woman (37 years with 13 cycles/years and two pregnancies, on average), the probability of having an episode of allopregnanolone withdrawal on a certain day is *p*_*woman*_ = 0.037. In men, data about neurosteroid withdrawal can be inferred from the seasonal variation of testosterone, showing two minima at spring and late summer [132]. Hence, for a man the probability of having an episode of neurosteroid withdrawal (notably androstanediol) on a certain day is *p*_*man*_ = 0.005. By multiplying these probabilities with the probability of having an excessively high cholesterol level on a certain day, the probability that the combination high-HPA/low-HPG activities will occur on that day would be obtained. With regard to stress, women develop psychosocial stress more often than men, but men respond to stress with higher cortisol rise [133]. Hence, as a first approximation it can be assumed that the frequency of episodes of excessively high cortisol potentially triggering FM is similar in both sexes. Therefore, the gender prevalence of FM predicted by our model would be $G=\frac{p_{woman}}{p_{man}}=6.8$, corresponding to 87.2% women among FM patients. This value is close to the average of the range of female prevalence reported for FM (about 80–90%) [11], thus being a satisfactory prediction of our model.

## Discussion

We have described a theoretical model of FM pathogenesis based on literature data. This new model includes elements from our previous hypothesis of a pain-processing thalamocortical loop [18], and our previous review of FM in pregnancy [21]. The model is supported by literature data as follows. First, it is consistent with the new concept of nociplastic pain [134,135], in which peripheral immune-endocrine signaling, combined with genetic predisposition and cognitive–emotional mechanisms, are thought to lead to structural and functional neuroplasticity in pain processing circuitry. Second, it fits data about the presence of GABA-glutamate unbalance [136–138], and the finding of glutamic acid decarboxylase decrease in FM patients [139]. Third, it is in tune with a bulk of data suggesting a role of HPA and HPG axes in FM pathogenesis [10–17], and with the influences exerted by these endocrine systems on brain networks (see above the sections: “*Influence of the HPG axis on the thalamocortical loop”* and *“Influence of the HPA axis on the thalamocortical loop”*). Fourth, it is compliant with the generally poor efficacies of drugs currently used in FM therapy, but also with the relatively higher efficacy of gabapentinoids [7,140]. Fifth, progesterone and testosterone, but not estradiol, seem to have a protective role in FM pain severity [33]. Finally, the model is a complete one, as it connects environmental stimuli and predisposing or constitutive conditions, such as sex, with a brain network that is thought to give rise to chronic pain, i.e. the main FM symptom. Such a connection is realized by a combined action of the HPG and HPA axes on the GABA/glutamate balance in the pain-processing loop. For a disease like FM, having puzzling etiology, unknown primary anatomical site, and lacking biological markers, the formulation of a complete model of pathogenesis is extremely relevant, because if the model holds, it also points to the most convenient targets to be addressed by therapeutic approaches.

The model predicts that the onset of FM is more likely during periods of low HPG axis activity (even though such activity does not necessarily remain low after the onset of the disease), consistent with epidemiological data showing a significantly higher incidence in women with suspected or proven infertility due to gynecological diseases. Some of these conditions, including menstrual disturbances, uterine disorders, ovarian dystrophy, ovarian cysts, miscarriage, and stillbirth, have been attributed, at least in some cases, to a depression of HPG functioning caused by excessive HPA activation [141].

Besides providing a possible explanation for FM pathogenesis, our model also provides a suitable prediction of female prevalence (see above the section: “*Predictivity and limitation of the model”*). This is consistent with premenstrual and prepartum allopregnanolone withdrawal in women caused by a significant scaling down of progesterone. In men, neurosteroid levels are also variable, but fluctuations are lower than in females [69]. Another possible cause of female prevalence resides in the reported estradiol positive modulation of NMDAR activity [142], thus acting opposite to progestin positive GABA_A_ modulation. In addition, an increase in the efficacy of ACTH as an HPA activator in women, and conversely a decrease in men, have been reported to occur from 30 to 60 years of age [143], coincident with the period of maximum FM incidence.

By providing indications about FM pathogenesis, our model also suggests therapeutic targets. Neurosteroids are expected to be the agents that mediate the effects of HPG and HPA axes on the central nervous system. Such a prediction is consistent with reported correlations between neurosteroids and a series of neurological disorders, including migraine, postpartum-depression, epilepsy, anxiety, cognitive and psychiatric disorders [56,144,145]. In this context, one of the most investigated condition is catamenial epilepsy, characterized by perimenstrual seizure exacerbation, involving altered levels of anticonvulsant THDOC, proconvulsant DHEA-S and cortisol [146], and premenstrual allopregnanolone withdrawal [147]. As another example, is PMDD is thought to derive from increased sensitivity to stress at the late luteal phase due to poor allopregnanolone-dependent GABA control of the HPA axis [148].

Even though the pathogenic mechanisms of these diseases are still unknown, a positive effect of neurosteroids in their treatment seems unquestionable, and accordingly, endocrine therapies have been considered [145]. Preclinical studies in a rat model of catamenial epilepsy have shown anticonvulsant effects of different neurosteroids acting as GABA_A_ PAM [149], while clinical trials have also been conducted with progesterone, the synthetic progestin norethisterone, and the gonadotropin antagonist goserelin [54,147]. GABA_A_ PAM neurosteroids have also anxiolytic and antidepressants properties, while their targets include a wider complex of GABA_A_ receptors (both γ-type and δ-type) with respect to benzodiazepines (γ-type only), possibly explaining the absence of antidepressant effects of these latter [150]. These neurosteroids are thought to be effective across a range of psychiatric disorders, including essential tremor and insomnia [150].

Among the most advanced neurosteroid-based treatments for central disorders, the synthetic analogue of allopregnanolone, brexanolone has been approved by the FDA for post-partum depression [151]. The analogous compound zuranolone (SAGE-217), also approved by FDA for the same disorder [152], has an increased oral bioavailability and limited side effects [153,154]. Its mechanism of action is presumed to involve an allopregnanolone-mimicking GABAergic modulation with regulatory effects on HPA, representing an intriguing convergence with our model.

For what concerns FM, both pharmacological and nonpharmacological therapies are generally disappointing, providing very limited benefit to FM patients [4,140,155,156]. Neurosteroids have never been considered for FM treatment, their closest use being the management of neuropathic pain [157]. Our model suggests a role for neurosteroids in FM pathogenesis, thus establishing a link between FM and several other central disorders with female prevalence. Therefore, our model indicates the use of natural or synthetic GABA_A_-active neurosteroids as a possible new therapeutic approach to FM. Considering the nonequivalent action of neurosteroids on GABA with respect to the effect of benzodiazepines, their therapeutic use deserves to be considered in future FM clinical trials. Although pain is the most characteristic FM marker, patients are affected by a cluster of symptoms that could be induced by the combined action of the HPG and HPA axes on other brain networks through parallel or cascade processes. In this view, the effectiveness of neurosteroids on migraine, anxiety, cognitive and psychiatric disorders, that very often accompany FM pain, is promising to improve patients’ quality of life.

Our model pertains to the problem of disease management, i.e. it is intended to fill the knowledge gap in FM investigations by proposing a unifying mechanism of pathogenesis. However, at present the model is a hypothesis that must be tested in future research. The lack of knowledge about FM pathogenesis hinders the development of molecular and cellular models, but a few symptomatic animal models have been realized in rodents. The most studied ones include stress-based methods, acid saline muscle application, and biogenic amine depletion by subcutaneous reserpine injection [158]. These models respond to pharmacological treatments having some effect on FM patients, like pregabalin and SNRIs [158], but the adopted mechanisms disregard brain networks, the role of HPG axis, and the interaction between HPA and HPG axes, thus being unsuitable as validating test of our model. Nevertheless, if combined with ovariectomy (low gonadal hormones), the stress-based models (presumably inducing high HPA axis activity) could become a possible experimental test for our theoretical model as a next investigation step.

The power of our FM model based on loop dynamics resides in the possibility of identifying putative therapeutic targets in biological correspondents of bifurcation parameters (see Box 1). This leaves room, at least in principle, for the possibility of going directly to clinical studies. Therefore, the use of the above-mentioned neurosteroid drugs in FM clinical trials could be a resolutive test of our model. This raises the problem of patient management, which in contrast to disease management must consider patient intervariability. Hence, while our disease-management model suggests neurosteroids as a promising pharmacological therapy, these drugs could be used in combination with non-pharmacological treatments. Such a strategy might help reduce the doses and the side effects of drugs, while being considered effective and appreciated by patients [4]. Evidence is increasing regarding the beneficial effects in FM of physical exercise, hyperbaric oxygen therapy (HBOT), and non-invasive brain stimulation (NIBS) [155,159,160]. Combining these therapies with neurosteroids could boost both structural and functional neuroplasticity and allow the recovery of dysregulated pain circuitry whilst improving the cluster of FM symptoms.

## Code availability

MATLAB codes have been made available online at https://zenodo.org/, DOI: 10.5281/zenodo.11445716.
